# Accuracy of real-time PCR and digital PCR for the monitoring of total HIV DNA under prolonged antiretroviral therapy

**DOI:** 10.1038/s41598-022-13581-8

**Published:** 2022-06-04

**Authors:** Constance Renault, Karine Bolloré, Amandine Pisoni, Camille Motto-Ros, Philippe Van de Perre, Jacques Reynes, Edouard Tuaillon

**Affiliations:** 1 Pathogenesis and Control of Chronic and Emerging Infections, University of Montpellier, INSERM, Etablissement Français du Sang, Antilles University, Montpellier, France; 2 CHU de Montpellier, Montpellier, France; 3 IRD UMI 233, INSERM U1175, Montpellier University, Montpellier, France; 4 Infectious Diseases Department, CHU de Montpellier, Montpellier, France

**Keywords:** Diagnostic markers, Retrovirus, Biological techniques

## Abstract

Total HIV DNA is a standard marker to monitor the HIV reservoir in people living with HIV. We investigated HIV DNA quantification accuracy by a real-time PCR kit (qPCR) and digital PCR (dPCR) method within the same set of primers and probes. Among 48 aviremic patients followed for up to 7 years with qPCR, the mean coefficient of variation of total HIV DNA between two successive measurements was 77% (± 0.42log_10_ HIVDNA copies/10^6^ PBMC). The total HIV DNA quantified by the two PCR methods has a high correlation (0.99 and 0.83, for 8E5 and PLHIV samples, respectively), but we observed better repeatability and reproducibility of the dPCR compared to the qPCR (CV of 11.9% *vs.* 24.7% for qPCR, p-value = 0.024). Furthermore, we highlighted a decay of the number of HIV copies in the 8E5 cell line qPCR standard over time (from 0.73 to 0.43 copies per cell), contributing to variations of HIV DNA results in patients whose HIV reservoir should be theoretically stabilized. Our study highlighted that absolute quantification of total HIV DNA by dPCR allows more accurate monitoring of the HIV reservoir than qPCR in patients under prolonged antiretroviral therapy.

## Introduction

Human immunodeficiency virus (HIV) provirus persists in the genome of latently infected cells despite suppression of viral replication by current antiretroviral therapies^1^. HIV reservoir is therefore considered a major obstacle to HIV cure. The quantification of total HIV DNA in whole blood or peripheral blood mononuclear cells (PBMC) by quantitative polymerase chain reaction (qPCR) is the standard method to estimate the HIV reservoir in people living with HIV (PLHIV)^2,3^. HIV DNA can be quantified in routine practice following treatment initiation and PLHIV under prolonged antiretroviral therapy^3^. The total HIV DNA quantification has been envisaged in French guidelines as a decision support tool for reducing the number of active ARVs^4^. Total HIV DNA includes integrated genomes that can be functional and non-functional forms (1 and 2 LTR-circles that are episomal genomes representing abortive integration events and linear forms), forming the total HIV DNA reservoir^3^. Studies have shown that the total HIV DNA is correlated with cells containing a replication-competent virus^3,5^. A rapid decay of HIV DNA is observed during the first year after the start of antiretroviral therapy^6^. Afterward, HIV DNA decay is slower or absent^2,6,7^. In people who achieve prolonged HIV suppression under antiretroviral treatment, the estimate of this marker of the viral reservoir is between less than 100 to 3000 HIV DNA copies per million PBMC^2,8^. Because of this relatively small concentration range, the quantification of HIV DNA requires a highly reliable method.

qPCR is based on the detection of target DNA relative to a standard and on the measurement of a fluorescence signal over time. qPCR is a sensitive method to detect low concentrations of virus nucleic acids^9^. However, instability of the standard curve, changes in amplification efficiencies, and measurement during an exponential amplification process contribute to limit the accuracy of DNA/RNA quantification using the qPCR method^10,11^. The reproducibility of qPCR for HIV DNA quantification may be insufficient for therapeutic purposes, especially for low HIV DNA values that exhibit the highest variations^12^. Digital PCR (dPCR) is a promising alternative to qPCR for HIV DNA quantitation^13–15^. dPCR is based on a limiting dilution, splitting the target DNA into multiple partitions. This approach eliminates the need for a standard curve and allows absolute quantification of the target. The amplification in each partition is detected at the end of the PCR, and the DNA concentration is calculated from the number of positive partitions. As an endpoint, the PCR method measures the nucleic acid concentrations through the ratio between positive and negative partitions. However, dPCR would be theoretically more reproducible^15^. The greater analytical precision of dPCR compared to qPCR makes theoretically possible the detection smaller variations in HIV DNA concentrations. Based on this, many authors recommend using dPCR rather than qPCR for quantifying the HIV reservoir^14,16^. Therefore, the limits of qPCR for HIV DNA quantification in clinical practice and the potential benefit of dPCR over qPCR for improved monitoring of HIV DNA needs to be evaluated.

In this study, (i) we analyzed variations of HIV DNA levels using qPCR during the long-term follow-up of outpatients with durable viral suppression; (ii) we developed a dPCR using a chamber-based system, dedicated to HIV DNA quantification according to the recommendations of the dMIQE Group^17^; (iii) we compared the two PCR methods to highlight the highest precision of dPCR for HIV DNA quantification; (iv) we showed that variations in HIV DNA content of the 8E5 batches of standard contribute to the low reproducibility of qPCR.

## Results

### Clinical monitoring of total HIV DNA using qPCR

**Figure 1:**
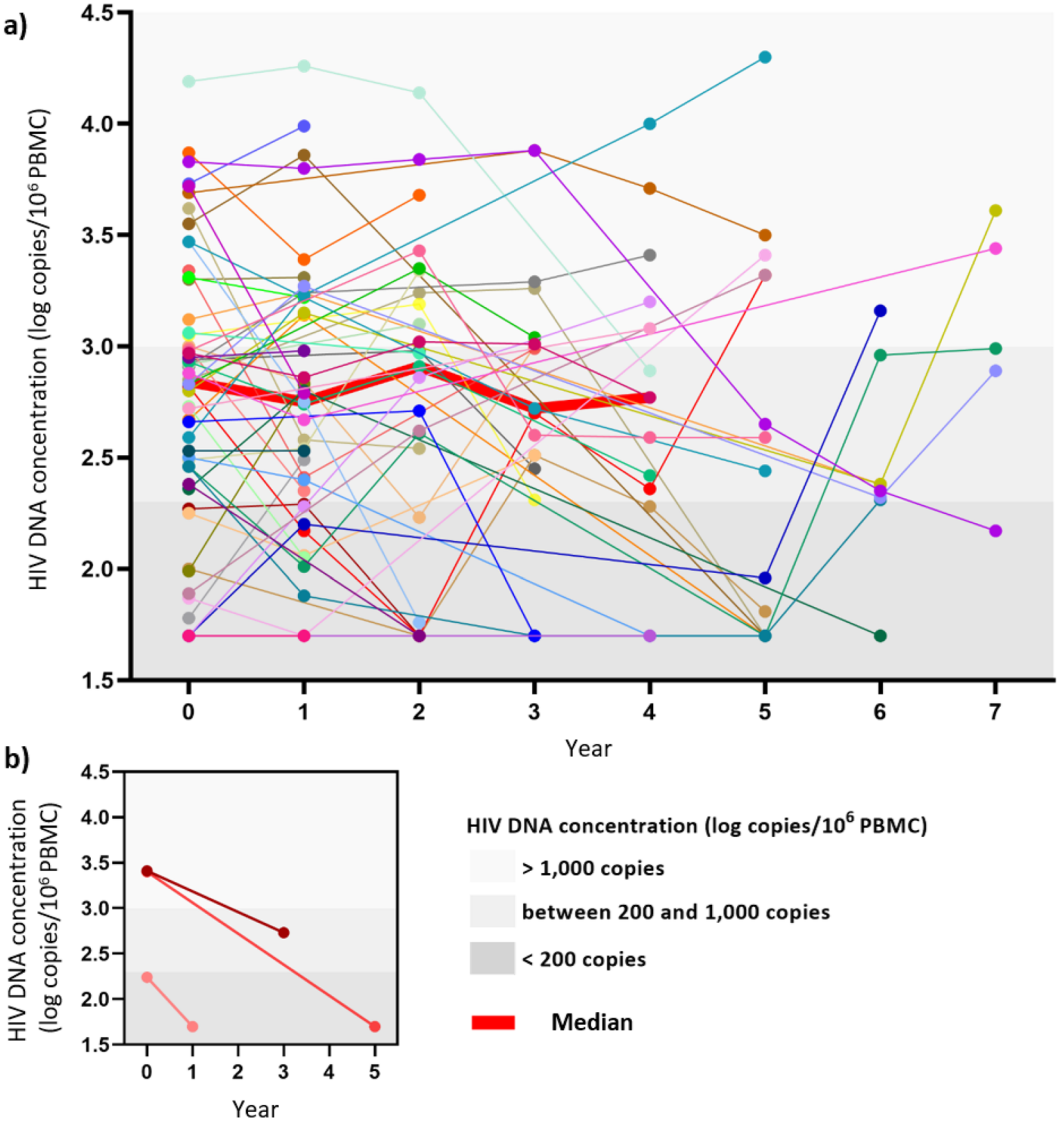
HIV reservoir follow-up study between 2014 and 2021. (**a**) HIV DNA quantification by qPCR from 2014 to 2021 in 48 PLHIV with prolonged virological success, where the y-axis shows the HIV DNA concentration (log_10_ copies/10^6^ PBMC copies), and x-axis the years (with 0 being the first quantification performed). (**b**) HIV-DNA quantification by qPCR in three patients initiating antiretroviral therapy.

Total HIV DNA was quantified in 48 subjects with prolonged virological success and 3 subjects initiating antiretroviral therapy using the commercial HIV DNA kit. Sequential measurements of HIV DNA were performed using 215 clinical samples collected between 2014 and 2021, with two to seven samples per subject. The mean coefficient of variation (CV) of HIV DNA concentration between all measurements of HIV DNA was 77%, corresponding to a mean variation of ± 0.42 log HIV-DNA copies/10^6^ PBMC between two successive measurements (Fig. 1a). A decrease in HIV DNA was observed for all three patients initiating antiretroviral therapy (Fig. 1b). In these subjects, the mean CV was 82%, corresponding to a decrease of 0.68 HIV DNA copies/10^6^ PBMC, one to five years after treatment initiation.

### Development of HIV DNA dPCR

The following criteria were used to select the optimal PCR conditions: the ratio of the mean fluorescence amplitude of HIV positive cell-line (8E5) to the mean fluorescence amplitude of PBMCs from uninfected controls, the visual separation of the positive and negative clouds allowing easy thresholding, and the number of false positives partitions present in PBMCs from uninfected controls. Different primers and probe concentrations, temperatures and time of hybridization and elongation, and numbers of cycles were tested to optimize the dPCR (Supplemental Fig. S1). An appropriate time of hybridization and elongation, and an appropriate number of cycles, were crucial to obtaining optimal discrepancy between positive and negative partitions. Different primer and probe concentrations were tested, ranging from concentration of 200 to 666 nM. For hybridization and elongation, we have chosen for optimal conditions a temperature of 57 °C for one minute, with 35 cycles where a concentration of 400 nM HIV DNA primers and 466 nM B-globin probe were retained in the PCR mix. The specificity was tested on ten human genomic DNA samples and ten clinical samples positive for different pathogens: hepatitis B virus, leptospirosis, and syphilis. No samples tested positive for HIV DNA (100% specificity, data not shown).

### dPCR analytical sensitivity

**Figure 2:**
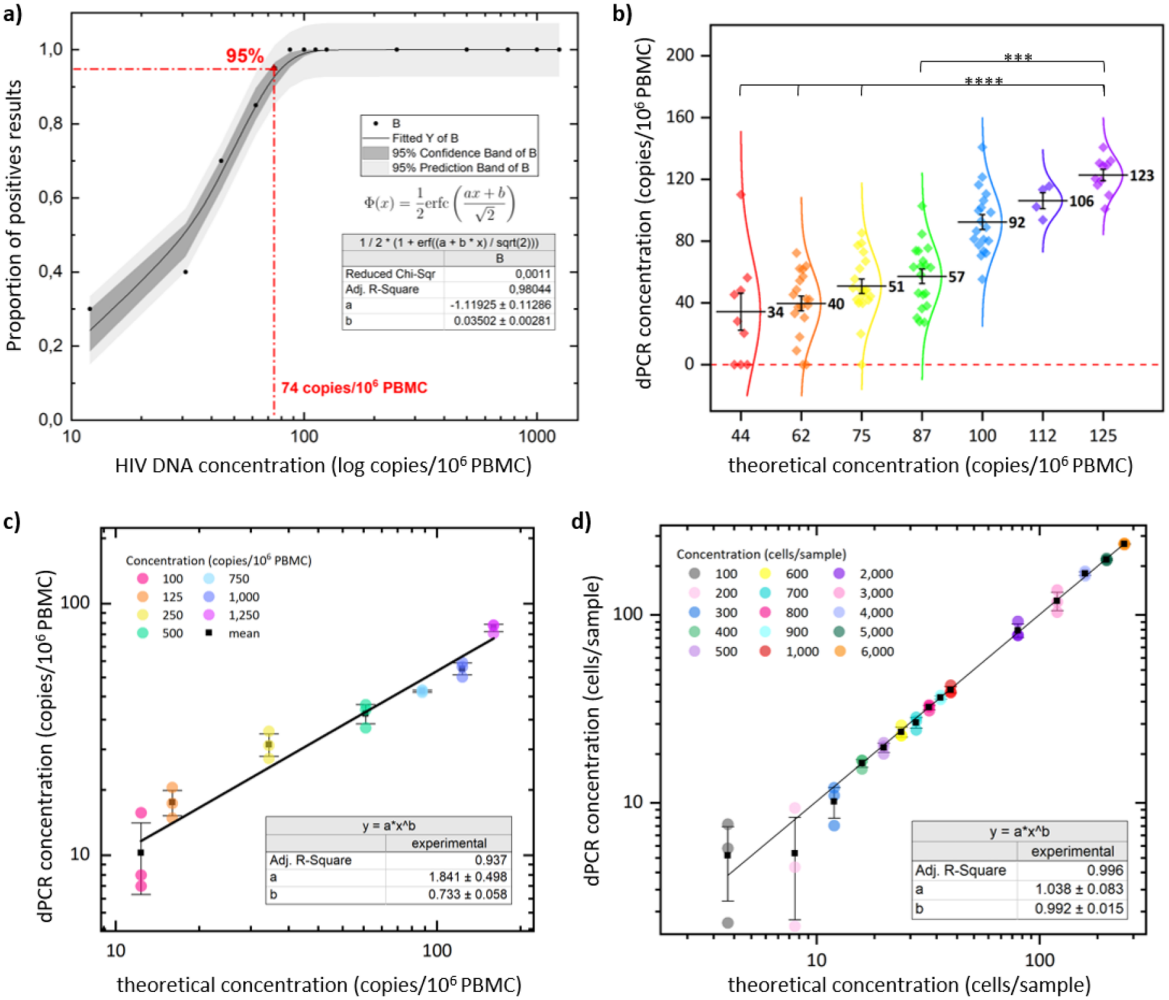
LOD95%, LOQ, and linearity study of the HIV DNA target in FAM and the β-globin target in VIC. (**a**) LOD95% of the target HIV-DNA in FAM is determined by a probit analysis at 74 copies/10^6^ PBMC. (**b**) shows dPCR replicates with concentrations ranging from 44 to 125 copies/10^6^ PBMC. The significant differences are indicated between the value of the LOQ and the other tested concentrations. (**c**) and (**d**) show linearity experiments where the data was fitted with a linear model, and, in (**d**), the concentrations ranged from 100 to 6000 cells/sample. On (**b**) and (**c**), the x-axis shows the theoretical concentration, whereas the y-axis shows the concentration measured by dPCR in copies/10^6^ PBMC, which allows the estimation of the LOD95% and LOQ for (**b**). The x-axis of (**a**–**d**) and the y-axis of (**b**–**d**) are displayed with a log scale.

We performed serial measurements in slightly varying concentrations to determine the limit of detection (LOD) and linearity of the HIV DNA dPCR. The 95% LOD was determined by testing 14 concentrations ranging from 12 to 1250 copies/10^6^ PBMC in multiple replicates (Table 1). The LOD95% result was estimated at the concentration of 75 copies/10^6^ PBMC, *i.e.* 0.6 copies/µL PCR, or 9 copies/replicate. The LOD95% determined by probit analysis was 74 copies/10^6^ PBMC (Fig. 2a). This concentration of HIV DNA was detected 19 times out of 20 replicates performed. The limit of blank was tested on 14 blank samples and estimated at 0.2 copies/µL PCR (data not shown). The established limit of quantification (LOQ) was 125 copies/10^6^ PBMC for this method with an accuracy of 98.4%, compared to the lower concentrations with 94.6% for 112 copies and 92.0% for 100 copies (Fig. 2b).

**Table 1:** LOD95% of the HIV DNA target in FAM.

Copies/10^6^ PBMC	Copies/PCR	Replicates	Positive results	% Detection
1250	15,000	10	10	100
1000	12,000	10	10	100
750	9000	3	3	100
500	6000	3	3	100
250	3000	3	3	100
125	1500	20	20	100
112	1350	5	5	100
100	1200	20	20	100
87	1050	20	20	100
**75**	**900**	**20**	**19**	**95**
62	750	20	17	85
44	525	10	7	70
31	375	10	4	40
12	150	10	3	30

The LOD95% experiment with each concentration tested indicated in copies/10^6^ PBMC and copies/PCR, the number of replicates performed, the number of positive results, and the percentage detection (positive results/total of replicates). The estimated LOD95% is shown highlighted in bold in the table.

To evaluate the linearity of the dPCR, we assessed a range of HIV DNA concentrations that included more than 75% of the theoretical values of the HIV reservoir in subjects with prolonged virological success^2^, i.e., between 100 to 1250 copies/10^6^ PBMC (Fig. 2c). The dPCR exhibited high linearity with an coefficient of determination (R^2^) close to 1 (R^2^ = 0.937).

We evaluated the analytical sensitivity of the dPCR for the β-globin target to determine if a low number of cells could be accurately detected and quantified by this technique. A range of 15 samples with a volume of 100 µL and concentrations ranging from 100 to 6000 cells per sample were measured in triplicates to test the LOD95% and LOQ of the β-globin dPCR (Fig. 2d). All concentrations tested were detected at 100%, so we did not determine a LOD95%, the lowest detected concentration being 100 cells/sample.The linearity experiment was then conducted on these same concentrations, demonstrating good linearity with an R^2^ very close to 1 (R^2^ = 0.996) for the β-globin target of digital PCR (Fig. 2d).

### dPCR technique is more accurate than qPCR

**Figure 3:**
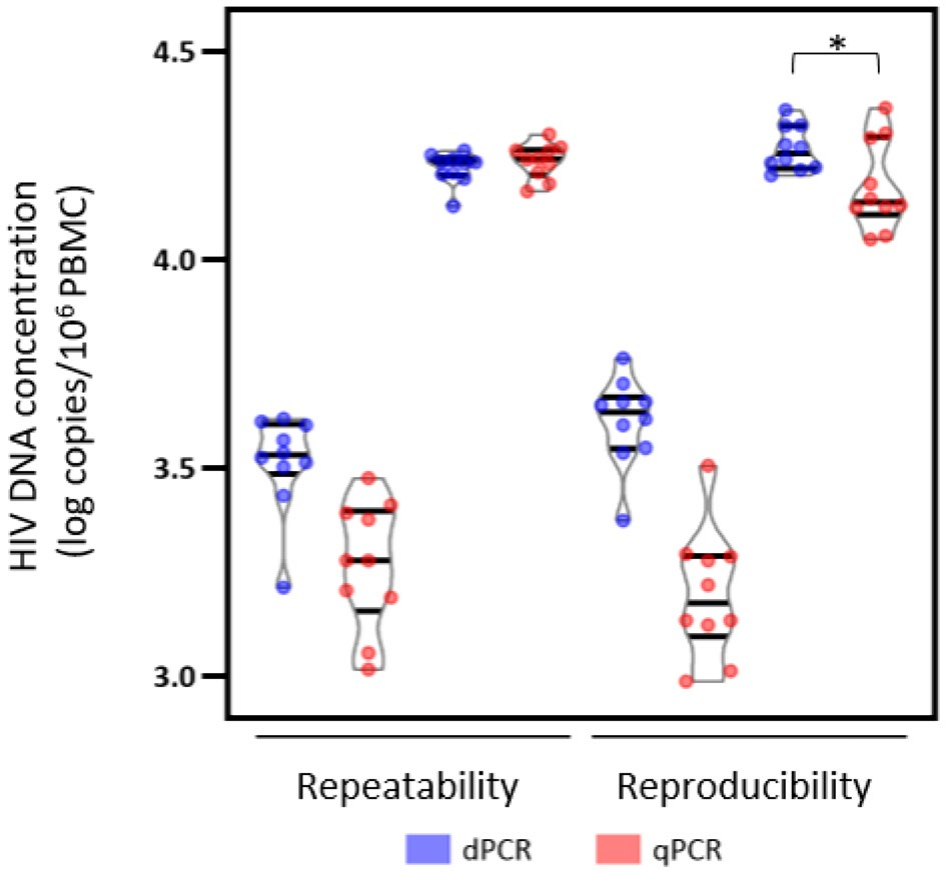
Precision experiments to compare the dPCR and qPCR methods with the coefficient of variation (CV%). The data of the repeatability and reproducibility studies for the two concentrations (100 and 1000 copies/10^6^ PBMC) are represented in log-transformed data in a violin plot. The violin plot shows median values and 25% and 75% interquartile ranges of the CT and 5% and 95% ranges. The non-parametric test (Siegel-Tukey) shows that the CV% of the reproducibility at 1000 copies/10^6^ PBMC is significatively different between the dPCR and qPCR with a p-value = 0.024.

At a concentration of 100 HIV DNA copies/10^6^ PBMC, the CV for dPCR was 21.29% for both the repeatability and reproducibility (Table 2a). By comparison, qPCR had a CV of 31.16% for repeatability and 36.79% for reproducibility. At 1000 copies/10^6^ PBMC, dPCR had a CV of 7.96% for repeatability and 11.92% for reproducibility (Table 2b), while qPCR showed a CV of 8.96% and 24.74% for repeatability and reproducibility, respectively. A non-parametric test (Siegel-Tukey) showed that the reproducibility at 1000 copies/10^6^ PBMC was significantly higher for the dPCR than qPCR (p-value = 0.024). Therefore, the dPCR had a more accurate inter-experimental precision than the qPCR (Fig. 3).

**Table 2:** Precision experiments to compare the dPCR and qPCR methods with the coefficient of variation (CV%).

(a) 100 copies/10^6^ PBMC		dPCR	qPCR
Repeatability	%CV	21.29	31.16
	%CV log_10_ *formula 1*	3.22	4.43
	%CV log_10_ *formula 2*	26.52	34.25
Reproducibility	%CV	21.29	36.79
	%CV log_10_ *formula 1*	2.80	4.55
	%CV log_10_ *formula 2*	23.63	34.47

(b) 1000 copies/10^6^ PBMC		dPCR	qPCR
Repeatability	%CV	7.96	8.96
	%CV log_10_ *formula 1*	0.87	0.93
	%CV log_10_ *formula 2*	8.42	9.12
Reproducibility	%CV	11.92	24.74
	%CV log_10_ *formula 1*	1.18	2.43
	%CV log_10_ *formula 2*	11.64	23.72

For the two concentrations, (**a**) 100 copies/10^6^ PBMC and (**b**) 1000 copies/10^6^ PBMC, the tables show the results of the repeatability and reproducibility studies in CV% in normal and log-transformed data calculated with the $formula \; 1=\sigma /\mu$ and the CV% of log-transformed data calculated with the $formula \; 2=\sqrt{{e}^{{\sigma }^{2}}-1}$. Formula 1 is commonly used to calculate the CV of untransformed data, and formula 2 the CV of log-transformed data. The CV calculation with formula 1 is often used in the literature. However, with a serious statistical error, Formula 1 is not correct for calculating the coefficient of variation of log-transformed data because the mean μ depends on the unit.

### Evaluation of HIV DNA content of the 8E5 cell-line batches used as a standard by the qPCR kit

**Figure 4:**
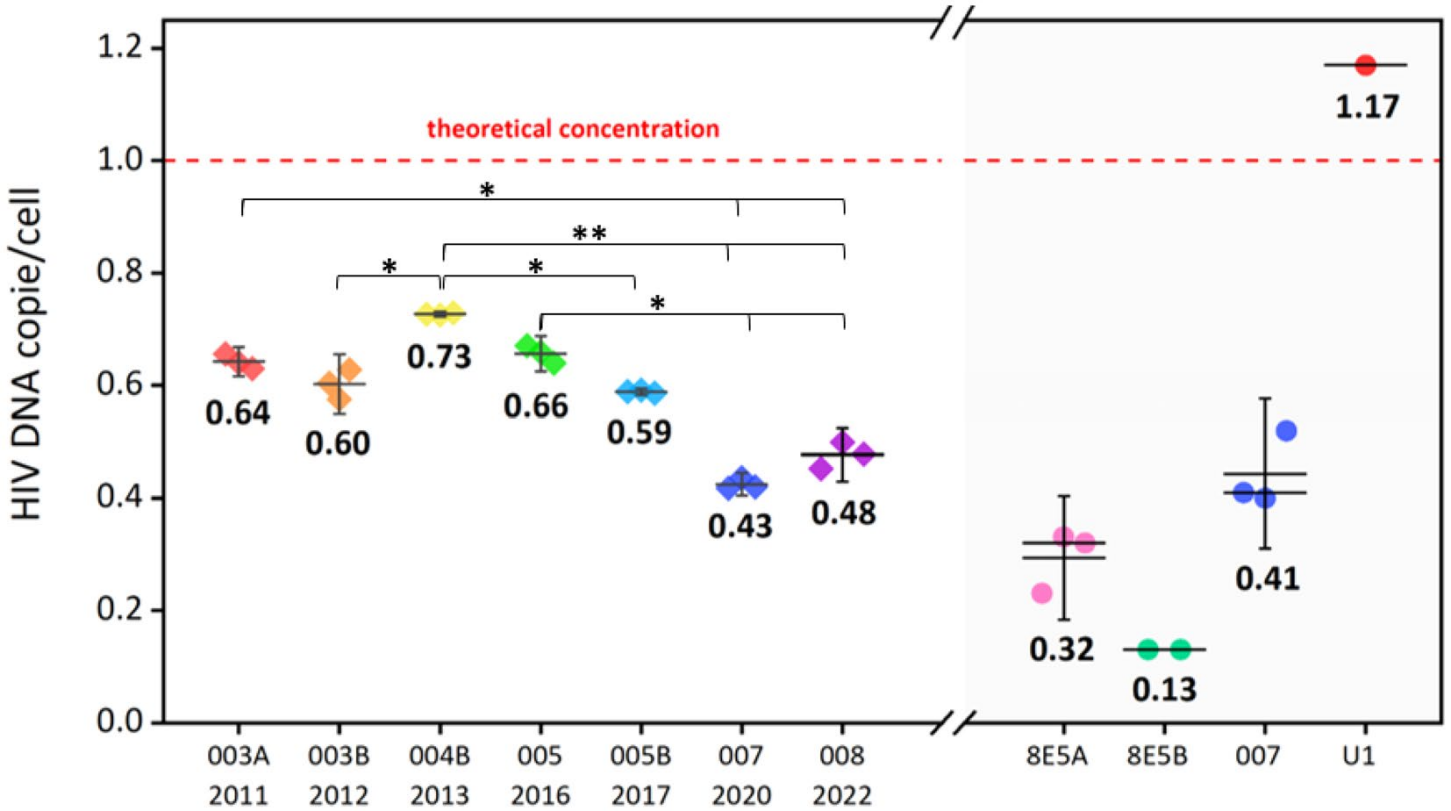
Standard test of the qPCR kit over several years and of different cell lines. The left part of the graph shows the quantification of HIV DNA performed in triplicates by the dPCR thermocycler ThermoFisher on the different batches of the Biocentric qPCR kit, where the y-axis represents HIV DNA copies/cells while the x-axis is the batch numbers and kits expiration dates. The right and shaded part of the graph shows the quantification of HIV DNA performed on three technologically different dPCR thermocyclers: ThermoFisher, Bio-Rad, and Stilla for the 8E5A cell-line and the standard 007; ThermoFisher and Bio-Rad for the 8E5B cell-line, and ThermoFisher for the U1 cell-line.

The 8E5 cell line standard from several batches of the qPCR kit was quantified using the dPCR to assess possible variations in HIV-1 DNA concentrations. Theoretically, the standard has a concentration of 1 copy of HIV DNA per cell or 3 × 10^6^ copies/mL^18^. Absolute quantification with dPCR showed lower concentrations in all the lots tested, ranging from 0.43 to 0.73 copies per cell (1.3 × 10^6^ to 2.2 × 10^6^ copies/mL). A progressive decrease in cellular HIV DNA was observed between successive lots over time (Fig. 4).

To confirm this difference, we quantified two 8E5 cell-lines stored in our laboratory (8E5A and 8E5B) and the 007 Lot standard on two additional dPCR thermocyclers, Bio-Rad and Stilla, in addition to the ThermoFisher thermocycler (Fig. 4). The laboratory cell line 8E5A was quantified at 1 copy of HIV DNA per cell by qPCR, but at 0.23, 0.32, and 0.33 copies per cell by dPCR performed on all three machines, respectively. The 8E5B cell-line was quantified at 0.13 copies per cell by the two dPCR thermocyclers, Bio-Rad and Stilla. The 007 Lot standard was quantified at 0.40, 0.41, and 0.52 copies per cell by dPCR. As a comparison, the ThermoFisher thermocycler quantified the chronically infected promonocytic cell line U1 at 1.17 copies per cell (Fig. 4)^19,20^. Therefore, the results obtained with several dPCR thermocyclers allowed us to confirm the deviation of the HIV DNA cell concentration of the standard used in qPCR from the theoretical value used as a reference.

### Comparison of total HIV DNA levels using dPCR and qPCR

**Figure 5:**
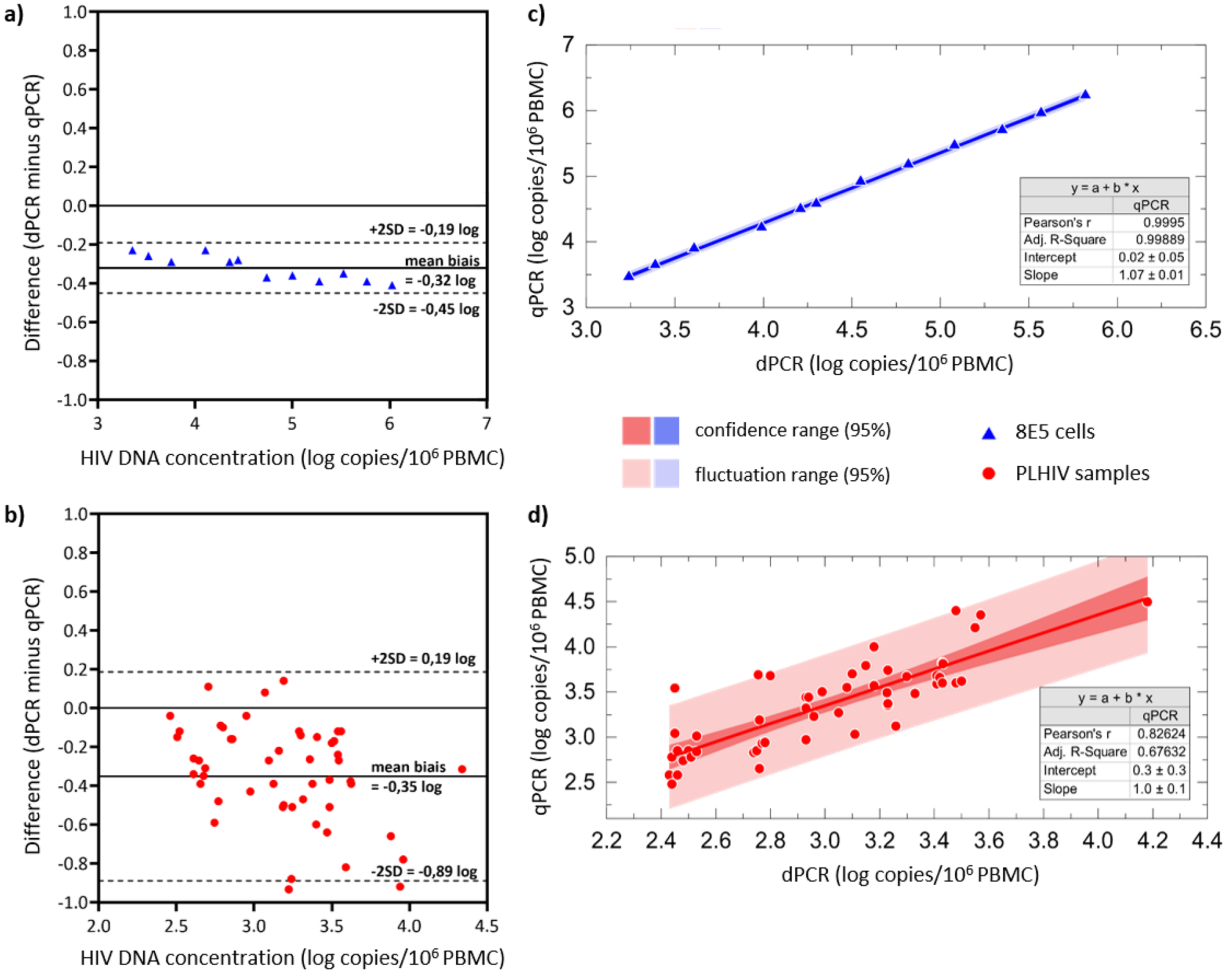
Comparison study between the dPCR and the qPCR. Bland–Altman dPCR minus qPCR was performed for (**a**) 12 samples of 8E5 cells, (**b**) 52 samples of PLHIV. Calculation of the correlation coefficient between the quantification values obtained with the qPCR and the qPCR for (**c**) 8E5 cells samples and (**d**) PLHIV samples.

Bland–Altman analysis was performed using 12 dilutions of the 8E5 cell line in HIV-negative PBMC (Fig. 5a) and 52 individual patient samples (Fig. 5b). The dPCR tended to have lower quantification than qPCR, with a median difference between the two techniques of -0.32 log_10_ HIV DNA copies/10^6^ PBMC for the 8E5 cell line (p-value = 0.0005) and -0.35 log_10_ HIV DNA copies/10^6^ PBMC for the clinical samples (p-value < 0.0001). However, the results obtained with the two techniques were highly correlated for the 8E5 cell line samples (r = 0.99) and the PLHIV samples (r = 0.83) (Fig. 5c,d).

## Discussion

Total HIV DNA is considered a clinically relevant marker for long-term monitoring of HIV reservoir in patients under antiretroviral therapy^3^. Quantification of total HIV DNA in PBMC or whole blood does not permit analyzing the HIV reservoir in tissue compartments, nor distinguishes between replication-competent and defective HIV genomes. However, this marker only requires a simple venous blood sample and a readily available PCR test^3^. Accuracy and sensitivity are required to analyze HIV DNA levels in the narrow range of concentrations observed in patients under prolonged antiretroviral therapy.

During the follow-up of patients under prolonged antiretroviral therapy in whom the HIV reservoir was theoretically stabilized, we observed an important variation of total HIV DNA using qPCR. The accuracy of the qPCR technique must be evaluated when taking into account the mean HIV DNA level, generally below 3 log_10_ copies HIV DNA/10^6^ PBMC. Therefore, a variation of ± 0.4 log_10_ limits the usefulness of the HIV DNA qPCR for therapeutic monitoring. The reproducibility of total HIV DNA quantification using this qPCR assay has been evaluated in previous studies. The reproducibility of HIV DNA by qPCR is better than viral outgrowth assays; however, qPCR is not precise enough to properly quantify HIV DNA^21–23^. Based on 69 testing in two patients followed for nine months, the standard deviation of HIV DNA with Biocentric PCR was 0.11 and 0.18 log_10_ with 95 CI intervals of ± 0.22 and 0.35 log_10_^18^. In this study, the qPCR of both calibrated curves and clinical samples were carried out in duplicate. Reproducibility across laboratories of this qPCR reported a 0.2 log_10_ standard deviation in 10 laboratories using three dilutions of HIV cell line (2.0, 2.8, and 3.4 log_10_ HIV DNA copies/10^6^ PBMC)^24^. This reproducibility of qPCR may be sufficient to monitor the rapid decay of total HIV DNA in patients initiating antiretroviral therapy, but a better accuracy is required to compare HIV DNA in CD4 subpopulations, or in different tissue compartments^25–27^.

Our results suggest that the instability of HIV DNA content in 8E5 cell line may significantly contribute to the inaccuracy and intra-individual variations observed in patients. We observed a progressive decay of HIV copy per cells over time as successive lots were used. Previous studies have reported that the 8E5 cell line, which theoretically has 1 copy of HIV DNA per cell, can ‘lose’ the provirus during multiple cell passages^28,29^. To date, the deletion or mutation mechanisms probably involved in this pheromone remain unknown^28,30^. Based on the result of HIV DNA content measured in the 007 8E5 batch (0.48 HIV DNA copies/cell), the standard would be the source of a 0.3 log_10_ difference in quantification between qPCR and dPCR. This gap in HIV DNA corresponded to the difference observed between the two PCR methods. Variations in HIV DNA content of the standard compromised the accuracy of results recorded during the patients over several years. The U1 cell line, containing on average two proviruses per cell, has been proposed as a possible alternative to 8E5 cells^30,31^, but using dPCR, we also observed a lower HIV DNA copy per U1 cell than expected. Furthermore, we observed that depending on the HIV DNA concentration range of a sample, the difference in quantification between qPCR and dPCR changed. In the samples with high HIV DNA concentrations, qPCR tends to overestimate the concentration while dPCR underestimates it. Technical variations due to the equipment, and induced by users, are responsible for uncertainty when using quantitative PCR^32^.

The dPCR developed in this study with the same set of primers and probes that qPCR offers significant advantages when compared to qPCR. First, dPCR enables HIV DNA quantification in absolute concentration; secondly, we observed a higher reproducibility using dPCR than dPCR; finally, the dPCR includes an internal cell control targeting the β-globin gene to quantify the number of cells tested. Because of its characteristics, dPCR is a more promising method than qPCR for the analysis of the HIV reservoir.

The LOD of the HIV DNA dPCR based on the same HIV genomic target (LTR) and the same set of primers and probe is slightly higher than the qPCR LOD (i.e., 75 *vs.* 40 copies/10^6^ PBMC, respectively). Considering the difference in quantification in favor of qPCR (+ 0.3 log_10_), the LOD value for dPCR is theoretically around 150 copies of HIV DNA/10^6^ PBMC in qPCR. After correction, this value remains below the threshold of 2.39 log_10_ (200 copies/10^6^ PBMC) established with the qPCR kit and indicates a low HIV reservoir and an optimal therapeutic response^2^. Hence, the sensitivity of dPCR may be sufficient to quantify the HIV reservoir in most patients under prolonged antiretroviral therapy^2,8^.

The dPCR may also be more appropriate to quantify HIV DNA in a limited number of cells because this method uses a small volume of eluate after extraction (4 µL) compared to qPCR (25 µL). This low volume facilitates multiplicate assays. The use of quantification replicates allows the results to be reported as a median, eliminating outliers through statistical analysis and improves the accuracy of the measurement. For example, on a 100 µL eluate after extraction of DNA from cells, dPCR allows us to experiment 25 times for only 4 times in qPCR. It is possible to collect and extract more significant volumes of blood to multiply the qPCR tests to improve sensitivity and accuracy, but this approach has limits in the case of the HIV reservoir explored in CD4 subpopulations and rare cells. Our results are in line with previous studies using dPCR for HIV DNA quantification^33–35^. dPCR techniques based on multiple targets could also further increase the accuracy of HIV DNA quantification^36–38^. Although dPCR assays allow the study of the HIV reservoir with more accuracy, standardized protocols are needed, and manufacturers should pursue regulatory approval for monitoring of HIV infected patients using dPCR kits^39,40^.

In conclusion, we observed critical intra-individual variations of HIV DNA when using the qPCR in aviremic subjects. We showed that both the poor reproducibility of qPCR and changes over time in the HIV-1 DNA concentration in the 8E5 standard fuel these variations. HIV DNA dPCR technique allows independence from two pitfalls of qPCR, namely real-time measurement from exponential amplification and quantification relative to a standard. The dPCR offers better performance to measure HIV DNA because of its reproducibility and absolute quantification of HIV DNA.

## Methods

### Study design

In the first part of the study, we evaluated the variation in HIV DNA concentration quantified with a commercial qPCR kit in subjects followed for HIV infection^41–43^. Intra-individual variation in HIV DNA concentration over time was assessed in subjects who had been aviremic for at least 24 months and whose total HIV DNA level was considered stabilized.

In the second part of the study, we evaluated the potential gain of using a dPCR technique compared to qPCR. For this purpose, we: (i) developed a dPCR technique and evaluated its characteristics and analytical performances according to the dMIQE guidelines^17^; (ii) evaluated the variations in HIV DNA concentrations in different batches of the 8E5 line used as a standard for qPCR to highlight possible variations in the qPCR standard that would contribute to the lack of precision of this technique; (iii) compared HIV DNA values obtained in dPCR and qPCR from 8E5 cells diluted with PBMC and from clinical samples.

### Patients

Outpatients consulting for HIV therapeutic monitoring in the infectious and tropical diseases department of the Montpellier University Hospital were included after having provided written informed consent to the use of their clinical and biological data for the study (DC-2011-1401). This study has received an approval from *Comité de Protection des Personnes Sud Méditerranée III* institutional ethics committee and followed the guidelines and regulations of the same institute (ID RCB no 2011-A01566-35, and DC-2011-1401). Blood samples from 51 PLHIH were analyzed in the HIV reservoir follow-up study. 48 patients were in a prolonged virological success (HIV reservoir considered stabilized), and 3 patients were considered positive controls (less than 24 months after the treatment initiation phase with an HIV reservoir considered not stabilized). Patient characteristics are presented in Table 3.

**Table 3:** Patient data from the HIV reservoir follow-up study.

Gender	Age	HIV date	ARV initiation	Last ARV treatment	Nadir CD4	Last measurement of					
						CD4	CD4%	CD8	CD8%	CD4/CD8	Lymphocytes
M	68	1986	1992	Abacavir, lamivudine + efavirenz	179	989	54	476	26	2.08	1831
M	68	1989	2000	Abacavir, lamivudine + efavirenz	104	832	47	425	24	1.96	1770
M	60	1985	1995	Abacavir, lamivudine + etravirine	422	422	35	434	36	0.97	1206
M	33	2012	2016	Bictegravir, emtricitabine, tenofovir alafenamide	393	621	38	637	39	0.97	1633
M	48	2011	2011	Bictegravir, emtricitabine, tenofovir alafenamide	290	1307	50	836	32	1.56	3150
M	49	2011	2013	Bictegravir, emtricitabine, tenofovir alafenamide	538	878	57	293	19	3.00	1540
M	52	2014	2014	Bictegravir, emtricitabine, tenofovir alafenamide	331	480	54	204	23	2.35	889
M	61	2011	2011	Bictegravir, emtricitabine, tenofovir alafenamide	133	690	41	824	49	0.84	1682
M	77	1999	1999	Bictegravir, emtricitabine, tenofovir alafenamide	310	983	47	858	41	1.15	2195
M	50	2013	2013	Bictegravir, emtricitabine, tenofovir alafenamide + doravirine, islatavir	226	640	42	760	47	0.89	1066
M	58	1989	2004	Bictegravir, emtricitabine, tenofovir alafenamide + etravirine	1282	1921	32	1261	21	1.52	6004
F	45	1986	1995	Cobicistat, elvitegravir, emtricitabine, tenofovir alafenamide	81	1130	42	1130	37	1.15	2530
M	50	2011	2011	Dolutegravir + emtricitabine, tenofovir disoproxil	245	543	50	293	27	1.85	1086
M	64	2012	2012	Dolutegravir, abacavir, lamivudine	330	609	42	478	33	1.27	1449
M	48	2002	2009	Dolutegravir, lamivudine	357	558	49	319	28	1.75	1139
M	50	2006	2011	Dolutegravir, lamivudine	309	556	30	889	48	0.63	1853
M	50	2010	2010	Dolutegravir, lamivudine	361	1303	60	586	27	2.22	2172
M	50	2002	2003	Dolutegravir, lamivudine	237	237	30	276	35	0.86	1595
M	50	2002	2009	Dolutegravir, lamivudine	319	765	36	935	44	0.82	2124
M	53	1993	2011	Dolutegravir, lamivudine	525	776	29	990	37	0.78	2676
M	54	2004	2009	Dolutegravir, lamivudine	293	795	41	737	38	1.08	1939
F	56	1986	1992	Dolutegravir, lamivudine	292	628	31	992	49	0.63	2025
M	60	1993	1997	Dolutegravir, lamivudine	143	888	61	247	17	3.60	1455
M	61	1990	1994	Dolutegravir, lamivudine	87	331	29	559	49	0.59	1141
F	62	1989	1992	Dolutegravir, lamivudine	239	1438	40	1078	30	1.33	3594
M	62	2004	2009	Dolutegravir, lamivudine	380	1003	27	1746	47	0.57	3714
F	65	1991	1998	Dolutegravir, lamivudine	38	765	36	617	29	1.24	2126
F	65	2005	2005	Dolutegravir, lamivudine	1002	1376	44	1001	32	1.37	3128
M	65	2013	2013	Dolutegravir, lamivudine	95	446	33	540	40	0.83	1350
M	65	2006	2008	Dolutegravir, lamivudine	481	682	40	281	17	2.43	1954
M	66	2013	2013	Dolutegravir, lamivudine	437	969	47	811	40	1.19	1820
M	67	1985	1997	Dolutegravir, lamivudine	123	810	33	1080	44	0.75	2454
M	67	2011	2011	Dolutegravir, lamivudine	175	475	35	339	25	1.40	1357
F	70	1997	1997	Dolutegravir, lamivudine	82	651	32	997	49	0.65	2035
M	71	1986	1997	Dolutegravir, lamivudine	187	679	39	575	33	1.18	1741
M	73	1994	1994	Dolutegravir, lamivudine	19	539	30	934	52	0.58	1797
M	79	1997	1997	Dolutegravir, lamivudine	386	773	50	386	25	2.00	1545
F	83	1987	1997	dolutegravir, lamivudine	313	515	31	697	42	0.74	1282
M	34	2011	2011	Emtricitabine, rilpivirine, tenofovir alafenamide	398	774	54	373	26	2.08	1433
M	47	1995	1997	Emtricitabine, rilpivirine, tenofovir alafenamide	319	789	48	608	37	1.30	1643
M	58	1988	1997	Emtricitabine, rilpivirine, tenofovir alafenamide	405	628	25	1079	43	0.58	2510
M	62	2001	2002	Etravirine + raltegravir potassique	174	627	51	332	27	1.89	1230
M	62	1999	2000	Lamivudine + dolutegravir	164	457	22	1123	54	0.41	2079
M	62	1993	1994	Lamivudine + dolutegravir	120	738	42	756	43	0.98	1757
F	74	1995	1997	Lamivudine + dolutegravir	231	1004	33	1004	33	1.00	3043
M	66	1989	1999	Maraviroc + dolutegravir, lamivudine	445	962	46	795	38	1.21	2091
M	56	2007	2008	Raltegravir potassium + emtricitabine, tenofovir disoproxil	164	515	44	503	43	1.02	1170
F	54	2013	2013	Tenofovir disoproxil, doravirine, lamivudine	552	988	48	556	27	1.78	2059
**M**	**44**	**2014**	**2014**	**Cabotegravir + rilpivirine**	**238**	**717**	**30**	**956**	**40**	**0.75**	**2390**
**M**	**45**	**2016**	**2016**	**Dolutegravir + emtricitabine, tenofovir disoproxil**	**231**	**516**	**42**	**418**	**34**	**1.23**	**1228**
**M**	**55**	**2016**	**2016**	**Emtricitabine, rilpivirine, tenofovir alafenamide**	**818**	**1012**	**57**	**497**	**28**	**2.04**	**1776**

The three values shown in the table with bold values are the positive controls.

### Cells samples

The 8E5 cells constituting the standard for the qPCR assay were used for dPCR and qPCR analytical performance analysis. Theoretically, cells from this cell line contain one copy of mutated HIV DNA per cell^44^. The laboratory’s 8E5 cell line (8E5A)(ATCC CRL-8993TM) was thawed and cultured in RPMI 1640 medium complete with 9% SVF and stored at 37 °C and 5% CO_2_. Positive cell samples containing 10^6^ 8E5 cells and 10^6^ PBMC and negative samples containing 10^6^ PBMC were performed using the Bio-Rad TC10TM automated cell counter. PBMCs were obtained from healthy blood donors and cryopreserved until used. DNA was extracted from these samples on the QIAcube from QIAGEN using the QIAamp® DNA Mini Kit protocol. Quantification with Nanodrop One confirmed their cellular concentration at 61.85 ng/µL of DNA (*i.e.*, an equivalent of 2 × 10^6^ cells) for HIV-positive samples and 33.88 ng/µL of DNA (*i.e.*, an equivalent of 10^6^ cells) for HIV negative samples. These samples were then aliquoted and stored at − 20 °C before being used for dPCR development and as positive and negative controls. To test the dPCR’s internal control, the negative samples were also used to create a dilution range (giving concentrations between 100 and 6000 cells in 100 µL, the final volume of eluate after DNA extraction).

Aliquots of an 8E5 cell line (8E5B) and a U1 cell line^19,20^, donated by the laboratory IRIM of CNRS (Montpellier) at a theoretical concentration of 1 copy per cell, were also quantified by dPCR.

As previously described, the Biocentric, Generic HIV DNA CELL kit standard used in qPCR comprises DNA extracted from this cell line^24^. Dilutions of this standard, provided at a concentration of 3 × 10^6^ copies of HIV DNA/mL, were used to evaluate the analytical performance of the two PCR methods. Serial dilutions were performed with the human genomic DNA solution, also provided in the kit, to obtain concentrations from 12 to 1250 copies/10^6^ PBMC. For each test performed, dilutions were made on the same day and stored as aliquots at − 20 °C.

### Sampling procedure

DNA extraction was performed on a 200 µL volume of fresh blood on the QIAcube automated system from QIAGEN following the QIAamp^®^ DNA Mini Kit protocol. The extracted DNA eluate was then assayed with Nanodrop One and stored at − 20 °C before PCR techniques.

### Precision study

Repeatability and reproducibility experiments were performed simultaneously on both methods to test and compare the accuracy of dPCR and the qPCR. 8E5 cells samples at concentrations of 100 and 1000 copies/10^6^ PBMC, consistent with the values generally found for the HIV reservoir in patients with prolonged therapeutic success, were used. One of these concentrations was close to the LOD at 100 HIV DNA copies/10^6^ PBMC (1.25 times the LOD of dPCR and 2.5 times the LOD of qPCR), and the other at 1000 copies/10^6^ PBMC (12.5 times the LOD of dPCR and 25 times the LOD of qPCR). For repeatability, the samples were tested ten times on a single run and by a single operator. In the reproducibility study, the two concentrations were tested twenty times on twenty different runs over ten days and by two operators.

The results of these precision experiments are presented by the coefficients of variation (CV%) calculated from the untransformed data (*formula* 1 = σ∕μ) and on the log^10^ transformed data, by applying two different formulas to calculate the CV of the latter (*formula* 1 = σ∕μ and *formula* 2 = √(*e*^(σ2)^ − 1)). Formula 1 is commonly used to calculate the CV of untransformed data, and Formula 2 the CV of log-transformed data. Calculating the CV of log-transformed data using formula 1 is often used in the literature but has severe statistical errors, as this formula is incorrect because the mean μ depends on the unit used. Thus, using formula 1 by transforming data into logs could make the coefficient of variation vary to almost zero. To compare with the results reported in the literature, the coefficients of variation according to these three cases have been indicated.

### Probes and primers

To closely compare between the dPCR and qPCR techniques, we have used the same pair of HIV DNA primers and probe targeting HIV LTR, already used in the literature^18^ and by Biocentric’s Generic HIV DNA CELL kit. The probe (5′-6-FAM AAGTAGTGTGTGCCC MGB-3′) targets a conserved consensus region within the 121 bp sequence (Thermo Fisher Scientific), sense (5′-GCCTCAATAAAGCTTGCC-3′) and anti-sense (5′-GGCGCCACTGCTAGAGATTTT-3′) primers (Eurofins Scientific).

The second primers/probe pair was used in dPCR, targeting the β-globin gene as an internal cell control. The VIC probe (5′-AAGGTGAACGTGGATGAAGTTGGTGG-3′) (Life technologies) and the sense (5′-GTGCACCTGACTCCTGAGGAGA-3′) and anti-sense (5′-CCTTGATACCAACCTGCCCAG-3′) (Thermo Fischer Scientific) yielded an amplicon of 102 bp. Primers and probe design were performed on Primer3 using the sequence of the human β-globin region on chromosome 11 (GenBank: U01317.1) found in the NCBI database.

### dPCR

The dPCR technique was developed on Thermofisher’s QuantStudioTM 3D Digital PCR System thermal cycler. Samples were prepared with the QuantStudio^®^ 3D Digital PCR Master Mix v2 using the QS 3D DPCR V2 20K CHIP 12-PACK and dispensed using the QuantStudio^®^ 3D Digital PCR Chip Loader. The dPCR mix had a final volume of 15 µL and consisted of 4 µL of DNA sample, 7.5 µL of Master Mix v2, HIV-DNA and β-globin probes at a concentration of 200 nM, HIV-DNA primers at 400 nM, and β-globin primers at 466 nM. After distribution, the chips were loaded onto the thermal cycler to undergo the following amplification program: an initial denaturation time of 10 min at 96 °C, followed by 35 cycles of hybridization and elongation of 2 min at 57 °C and 30 s at 98 °C, then a final elongation time at 60 °C for 2 min. The chips were transferred and read on the QuantStudio chip reader at the end of this program. The dMIQE guidelines were followed to validate our digital PCR technique^17^.

Bio-Rad and Stilla digital PCR thermocyclers were used to test the Biocentric qPCR range. The PCR mix and program used on these dPCRs were the same as those developed on the QuantStudio 3D dPCR.

### qPCR from Biocentric

qPCR was performed following the protocol of the Biocentric Generic HIV DNA CELL kit on the Roche Life Science LightCycler^®^480^18^. According to the manufacturer and previous study the LOD was 40 copies/10^6^ PBMC^18^.

### Data analysis

qPCR results were analyzed on LightCycler^®^480 SW 1.5 software from Roche Life Science. dPCR results were analyzed on QuantStudio 3D Analysis Suite Cloud Software v3.1 from Thermo Fisher ConnectTM. As the automatic fluorescence threshold setting proposed by this software was not adapted to the analysis of a set of samples, several control points were put in place to perform a manual threshold setting^45,46^. Firstly, the chip had to be of high quality (no bubbles or reading problems) and have a total number of partitions greater than 15,000 out of the 20,000 possible partitions. A minimum threshold fluorescence level was established through simplex dPCR experiments for each target, on FAM fluorescence targeting HIV DNA and VIC fluorescence targeting the internal control β-globin. An assessment of the percentage of false positives was performed on a range of negative samples, allowing us to estimate the possible number of false positives at each threshold value^47^. A threshold of 2300 was chosen, giving a 0% median false positive (data not shown). With this minimum threshold and the study estimating the percentage of false positives at each threshold value, we manually choose the most appropriate threshold for each sample. Finally, the experiments were also analyzed in triplicates with a time-lapse between each analysis. Statistical analyses and figures were made on GraphPad Prism 8 and Inkscape.

## Supplementary Information

Supplementary Figure S1.
