# Supplementary material for: Accuracy of real-time PCR and digital PCR for the monitoring of total HIV DNA under prolonged antiretroviral therapy
Source: Sci Rep. 2022 Jun 4;12:9323. doi: 10.1038/s41598-022-13581-8 (PMC9167282; doi:10.1038/s41598-022-13581-8)
Supplement: Supplementary file 1 — Supplementary Figure S1. [file 41598_2022_13581_MOESM1_ESM.docx]

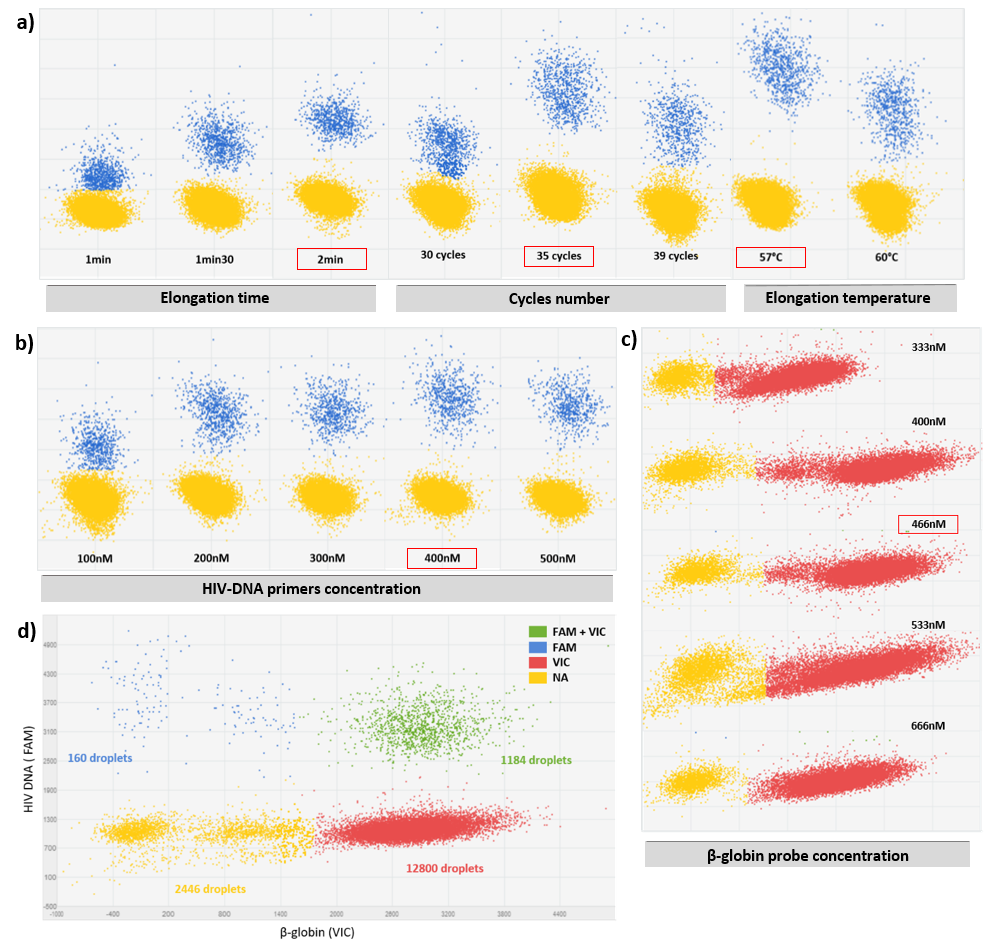


**Supplemental Figure S1. Development of the HIV-1 DNA and β-globin duplex PCR**. Optimization of **a)** the number of cycles, elongation time and temperature of the digital PCR program, **b)** the concentration of HIV DNA primers in the PCR mix, **c)** the concentration of the internal control β-globin probe. The red boxes show the choices made for each of these criteria. **d)** Example of a plot obtained with digital duplex PCR.
